# *Schinus terebinthifolia* Raddi. Leaf Lectin (SteLL) Demonstrates Anxiolytic and Antidepressant Effects Under Monoaminergic Deficiency Induced by Reserpine

**DOI:** 10.3390/plants14193048

**Published:** 2025-10-01

**Authors:** Bárbara Raíssa Ferreira de Lima, Leydianne Leite de Siqueira Patriota, Amanda de Oliveira Marinho, Thiago Lucas da Silva Lira, Jainaldo Alves da Costa, Beatriz Galdino Ribeiro, Daniella Carla Napoleão, Jorge Vinícius Fernandes Lima Cavalcanti, Michelly Cristiny Pereira, Moacyr Jesus Barreto de Melo Rego, Maira Galdino da Rocha Pitta, Thiago Henrique Napoleão, Michelle Melgarejo da Rosa, Patrícia Maria Guedes Paiva

**Affiliations:** 1 Departamento de Bioquímica, Centro de Biociências, Universidade Federal de Pernambuco, Recife 50670-901, Brazil; barbara.flima@ufpe.br (B.R.F.d.L.); leydianne.patriota@ufpe.br (L.L.d.S.P.); amanda.marinho@ufpe.br (A.d.O.M.); thiago.silvalira@ufpe.br (T.L.d.S.L.); jainaldo.costa@ufpe.br (J.A.d.C.); moacyr.rego@ufpe.br (M.J.B.d.M.R.); maira.pitta@ufpe.br (M.G.d.R.P.); patricia.paiva@ufpe.br (P.M.G.P.); 2 Departamento de Engenharia Química, Centro de Tecnologia e Geociências, Universidade Federal de Pernambuco, Recife 50670-901, Brazil; beatriz.galdino@ufpe.br (B.G.R.); daniella.napoleao@ufpe.br (D.C.N.); jorgevcavalcanti@gmail.com (J.V.F.L.C.); 3 Núcleo de Pesquisa em Inovação Terapêutica Suely Galdino (NUPIT), Universidade Federal de Pernambuco, Recife 50670-901, Brazil; michelly.pereira@ufpe.br; 4 Departamento de Fisiologia e Farmacologia, Centro de Biociências, Universidade Federal de Pernambuco, Recife 50670-901, Brazil

**Keywords:** depression, anxiety, plant lectin, monoaminergic depletion, neuroinflammation

## Abstract

*Schinus terebinthifolia* Raddi. leaf lectin (SteLL) has been investigated for its neuromodulatory effects. Given the etiological diversity of depression, this study evaluated the effects of SteLL in a pharmacological model induced by reserpine. Mice were administered reserpine intraperitoneally for 10 days to induce anxiety- and depression-like symptoms. Before reserpine administration, animals also received SteLL (2 or 4 mg/kg, i.p.) or fluoxetine (10 mg/kg, i.p.) for 10 days. Behavioral assessments included the open field test, elevated plus maze, and tail suspension test. Body weight variation and brain levels of cytokines, noradrenaline, dopamine, and serotonin were also analyzed. In reserpine-treated mice, SteLL administration (2 and 4 mg/kg) produced anxiolytic-like effects in the open field (reduced number of rearings) and elevated plus maze (increased time spent in open arms) and significantly reduced immobility time in the tail suspension test. Additionally, SteLL prevented the body weight loss typically induced by reserpine. SteLL treatment modulated neuroinflammation by reducing IL-2 and increasing IL-10 levels in the brain. SteLL treatment restored dopaminergic and noradrenergic levels, with no effect on serotonin. In conclusion, SteLL was effective in reserpine-induced monoaminergic depletion, reversing behavioral and biochemical alterations characteristic of depression, likely through dopaminergic, noradrenergic, and anti-inflammatory mechanisms.

## 1. Introduction

Anxiety is a natural human response to perceived threats or danger [1]. However, when it becomes disproportionate or persistent, it can develop into a pathological condition, affecting approximately 30% of adults at some point in their lives [2]. Similarly, depressive disorder is a debilitating psychiatric condition characterized by persistent sadness, anhedonia, cognitive dysfunction, and, in severe cases, suicidal ideation [3].

According to the World Health Organization [4], the global incidence of anxiety and depression increased by 25% during the first year of the COVID-19 pandemic. Although both disorders are treatable, current pharmacological interventions remain suboptimal, often associated with a delayed onset of action and significant side effects. Moreover, the multifactorial nature of these conditions further complicates the development of effective and targeted therapies [5,6]. In this context, natural products have garnered increasing attention as promising alternatives for the treatment of mood disorders, due to their diverse bioactive properties and generally low toxicity [3,7]. Among these, plant lectins—proteins with specific and reversible carbohydrate-binding capabilities—have demonstrated potential in modulating biological processes relevant to neuropsychiatric conditions [8].

*Schinus terebinthifolia* Raddi (Anacardiaceae), commonly known as the Brazilian pepper tree, is well known for its ethnomedicinal uses. Traditionally, bark-and-leaf tea is consumed as an antidepressant [9], while leaf decoctions are inhaled to relieve symptoms of depression [10]. Within this context, SteLL, a lectin isolated from the leaves of *S. terebinthifolia*, has demonstrated notable mood-regulating properties. SteLL (1 and 5 mg/kg, i.p.), administered for 7 consecutive days to tumor-bearing mice, did not cause mortality, produce alterations in blood biochemical or hematological parameters, or induce genotoxicity in bone marrow cells [11]. Furthermore, an acute toxicity assay using a higher dose of SteLL (100 mg/kg), administered either intraperitoneally or orally, showed no mortality, no signs of intoxication, no histopathological damage to the liver, spleen, kidneys, or stomach, and no genotoxic effects [12].

Lima et al. [13] showed that SteLL produces anxiolytic effects in mice, as observed through behavioral changes in the open field and elevated plus maze tests. These anxiolytic effects appear to be mediated primarily through monoaminergic pathways rather than the lectin’s carbohydrate-binding domain. In a subsequent study, SteLL was found to reduce immobility time in the tail suspension test, indicating antidepressant-like activity in mice. This antidepressant effect was dependent on the carbohydrate-recognizing domain of SteLL and involved both monoaminergic and nitric oxide signaling pathways [14]. More recently, SteLL was reported to alleviate anxiety- and depression-like behaviors in mice exposed to chronic stress. These improvements correlated with increased brain monoamine levels, reduced oxidative stress, and modulation of inflammatory cytokines [15].

While these results are encouraging, the etiological heterogeneity of depression necessitates rigorous validation of therapeutic effects across diverse experimental models. Therefore, the present study aimed to both validate and extend previous findings by investigating the effects of SteLL in a pharmacological model of depression induced by reserpine. Reserpine depletes monoaminergic neurotransmitters, replicating a key neurochemical characteristic observed in depressed patients. This model provides a complementary approach to further assess SteLL’s therapeutic potential and evaluate whether its antidepressant effects remain effective in the face of monoaminergic deficiency. Through this approach, we seek to reinforce the robustness of earlier data while deepening the understanding of SteLL’s neuromodulatory properties.

## 2. Results

### 2.1. Purification of SteLL

**Figure 1:**
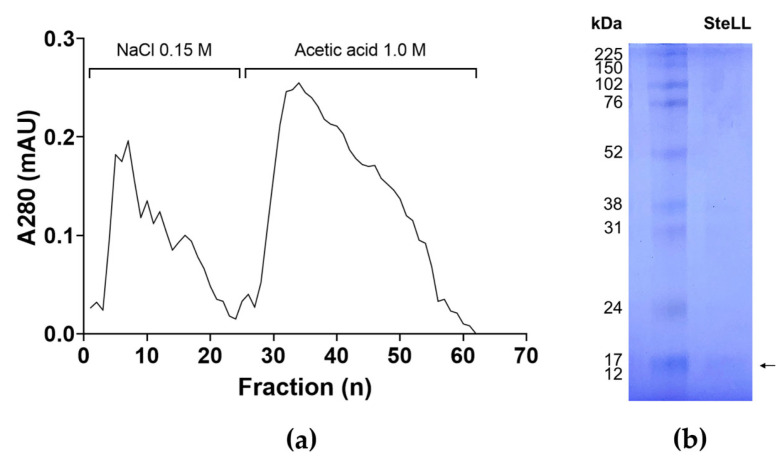
Purification of *Schinus terebinthifolia* leaf lectin (SteLL). (**a**) Chromatography of leaf extract on the chitin column. SteLL was eluted with 1.0 M acetic acid. (**b**) Polyacrylamide gel electrophoresis of SteLL (arrow) and molecular mass marker. The gel was stained with Coomassie Brilliant Blue.

The purification of SteLL from the leaf extract was performed according to previously established protocol. The lectin was eluted in the fractions adsorbed to the chitin column (Figure 1a), displaying with a specific hemagglutinating activity of 7224 units/mg. To confirm the reproducibility of the purification procedure, SDS-PAGE analysis was conducted, revealing a single polypeptide band of approximately 14 kDa (Figure 1b), consistent with findings reported by Gomes et al. [16] and subsequent studies [11,14,17]. A yield of 7.7 mg of SteLL was obtained from each gram of leaf powder.

### 2.2. Administration of SteLL Displayed Anxiolytic-Like Effects in Reserpine-Treated Mice

**Figure 2:**
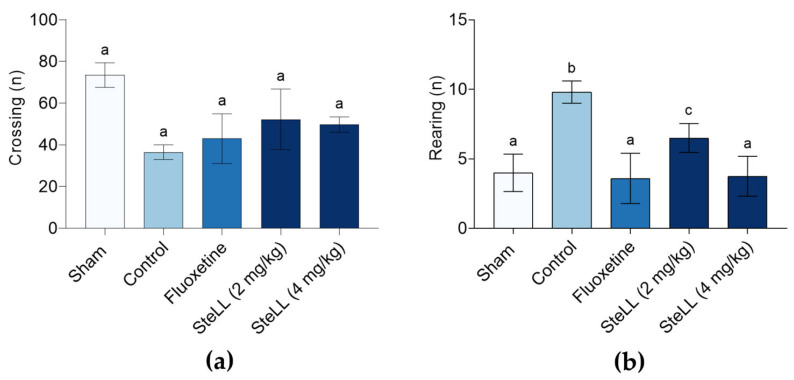
Effects of *Schinus terebinthifolia* leaf lectin (SteLL) in reserpine-treated mice evaluated in the open field test (OFT). The number of crossing (**a**) and rearing (**b**) actions by mice were recorded. Reserpine-treated animals received intraperitoneal injections of PBS (control), fluoxetine (10 mg/kg) or SteLL (2 or 4 mg/kg). The Sham group did not receive reserpine administration and was treated with PBS i.p. Data are expressed as mean ± standard error of the mean, SEM (n = 5). Bars labeled with different letters represent statistically significant differences between groups (*p* < 0.05), whereas bars sharing the same letter indicate no significant difference (*p* > 0.05), as determined by ANOVA followed by Bonferroni post hoc test.

The open field test (OFT) and the elevated plus maze (EPM) were employed to evaluate exploratory behavior and anxiety-like responses. In the OFT, analysis of the number of crossings (Figure 2a; F_4,20_ = 2.367, *p* = 0.0994) showed no significant differences among the control, Sham and groups treated with SteLL (2 and 4 mg/kg i.p.) or fluoxetine (positive control, 10 mg/kg i.p.). Regarding rearing behavior (Figure 2b; F_4,20_ = 4.089, *p* = 0.0157), the Sham group, both SteLL-treated groups, and the fluoxetine group exhibited a significant reduction in the number of rearings compared to the control group, indicating that both the lectin and the reference drug attenuated the anxiety-like behavior induced by reserpine. In addition, the rearing behavior of animals treated with fluoxetine and SteLL at 4 mg/kg was similar to that of the Sham group.

**Figure 3:**
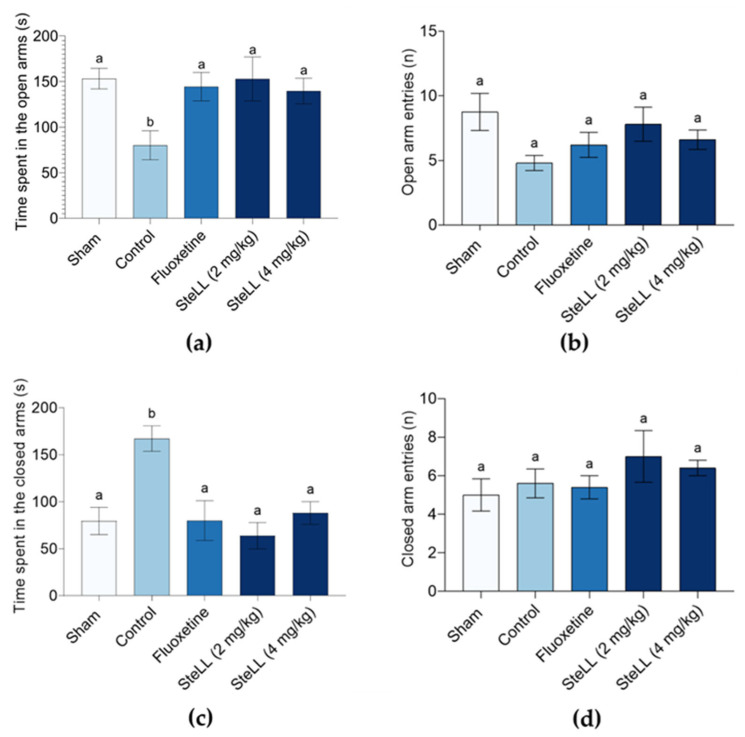
Effects of *Schinus terebinthifolia* leaf lectin (SteLL) in reserpine-treated mice evaluated in the elevated plus maze (EPM) test. The letters in the figure mean the time spent in open arms (**a**) and closed (**c**) arms as well as the number of arm entries in open (**b**) and closed (**d**) arms. Reserpine-treated animals received intraperitoneal injections of PBS (control), fluoxetine (10 mg/kg) or SteLL (2 or 4 mg/kg). The Sham group did not receive reserpine administration and was treated with PBS i.p. Data are expressed as mean ± SEM (n = 5). Bars labeled with different letters represent statistically significant differences between groups (*p* < 0.05), whereas bars sharing the same letter indicate no significant difference (*p* > 0.05), as determined by ANOVA followed by Bonferroni post hoc test.

In the EPM test, none of the treatments significantly altered the number of entries into either the open (Figure 3b; F_4,20_ = 2.078, *p* = 0.1239) or closed (Figure 3d; F4,20 = 0.9106, *p* = 0.4768) arms. However, animals treated with fluoxetine or both doses of SteLL, and the Sham group spent significantly more time in the open arms compared to the control group (Figure 3a; F_4,20_ = 3.356, *p* = 0.029), indicating lower anxiety-like behavior. Fluoxetine and SteLL restored the animals’ behavior to levels equivalent to those of the Sham group. Consistently, both SteLL or fluoxetine-treated groups spent significantly less time in the closed arms (Figure 3c; F_4,20_ = 7.176, *p* = 0.0011) than the control group, effectively restoring the animals’ behavior to levels equivalent to those of the Sham group.

### 2.3. SteLL Reduced Immobility Time in the Tail Suspension Test (TST)

**Figure 4:**
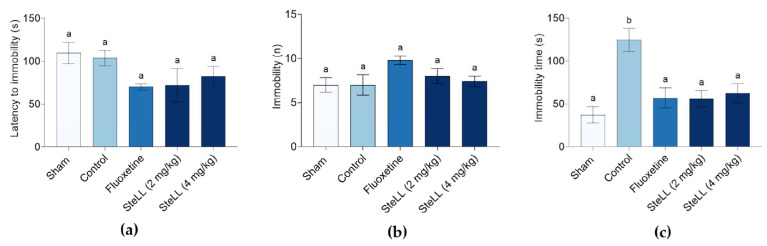
Effects of *Schinus terebinthifolia* leaf lectin (SteLL) in reserpine-treated mice evaluated in the tail suspension test (TST). Immobility latency (**a**), number of immobility episodes (**b**), and immobility time (**c**) were recorded. Reserpine-treated animals received intraperitoneal injections of PBS (control), fluoxetine (10 mg/kg) or SteLL (2 or 4 mg/kg). The Sham group did not receive reserpine administration and was treated with PBS i.p. Data are expressed as mean ± SEM (n = 5). Bars labeled with different letters represent statistically significant differences between groups (*p* < 0.05), whereas bars sharing the same letter indicate no significant difference (*p* > 0.05), as determined by ANOVA followed by Bonferroni post hoc test.

The TST was used to assess depression-like behaviors following the reserpine protocol. No significant differences were observed among the groups in latency to immobility (Figure 4a; F_4,20_ = 1.958, *p* = 0.1421) or in the number of immobility episodes (Figure 4b; F_4,20_ = 2.073, *p* = 0.1225). However, treatments with SteLL (2 and 4 mg/kg) or fluoxetine significantly reduced total immobility time compared to the control group (Figure 4c; F_4,20_ = 8.828, *p* = 0.0003), with values similar to those observed in the Sham group.

### 2.4. SteLL Treatment Prevented the Weight Loss Effects Caused by Reserpine Administration

**Figure 5:**
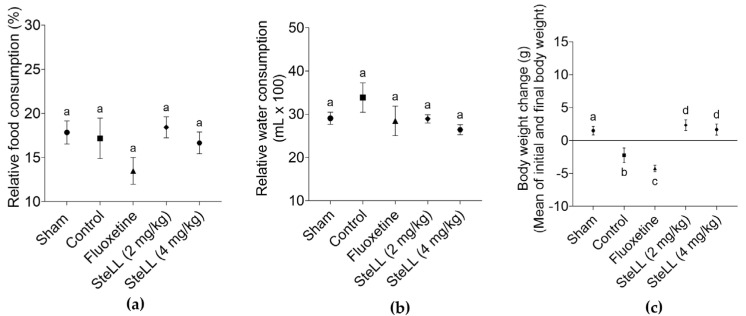
Relative water consumption (**a**), relative food consumption (**b**) relative water consumption, and (**c**) body weight variation in reserpine-treated animals that received intraperitoneal injections of PBS (control), fluoxetine (10 mg/kg) or SteLL (2 or 4 mg/kg). The Sham group did not receive reserpine administration and was treated with PBS i.p. Data are expressed as mean ± SEM (n = 5). Bars labeled with different letters represent statistically significant differences between groups (*p* < 0.05), whereas bars sharing the same letter indicate no significant difference (*p* > 0.05), as determined by ANOVA followed by Bonferroni post hoc test.

Relative consumption of water (Figure 5a; F_4,20_ = 1.359, *p* = 0.2834) and food (Figure 5b; F_4,20_ = 1.528, *p* = 0.2322) were not different between the groups. Animals treated with SteLL and from the Sham group showed significant body weight gain, while body weight loss was found for control and fluoxetine-treated group (Figure 5c; F_4,20_ = 11.29, *p* < 0.0001), suggesting a remarkable protective effect of the lectin against the catabolic effects induced by the reserpine protocol. The body weight gain in the groups treated with SteLL was even higher than that observed in the Sham group.

### 2.5. Effect of SteLL on Brain Cytokine Levels in the Reserpine Model

**Figure 6:**
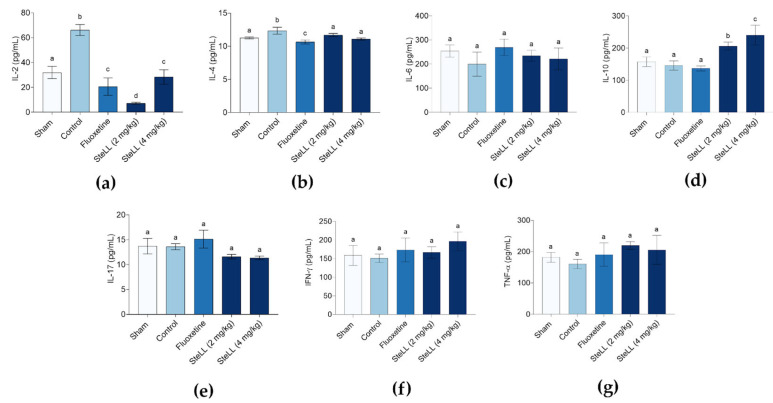
Effects of *Schinus terebinthifolia* leaf lectin (SteLL) on brain cytokine levels in reserpine-treated mice. Animals received intraperitoneal injections of PBS (control), fluoxetine (10 mg/kg), or SteLL (2 or 4 mg/kg). The sham group did not receive reserpine and was treated with PBS. The levels of the following cytokines were measured: (**a**) IL-2; (**b**) IL-4; (**c**) IL-6; (**d**) IL-10; (**e**) IL-17; (**f**) IFN-γ; and (**g**) TNF-α. Data are expressed as mean ± SEM (n = 5). Bars labeled with different letters represent statistically significant differences between groups (*p* < 0.05), whereas bars sharing the same letter indicate no significant difference (*p* > 0.05), as determined by ANOVA followed by Bonferroni post hoc test.

Figure 6 shows that reserpine administration induced an inflammatory profile, as evidenced by elevated pro-inflammatory cytokine interleukin (IL) 2 levels in the control group, compared to the Sham group. Treatment with SteLL (2 and 4 mg/kg) or fluoxetine significantly reduced the IL-2 levels (Figure 6a; F_4,20_ = 18.69, *p* < 0.0001), with the 2 mg/kg dose of SteLL showing the most pronounced effect. The IL-4 level was significantly higher in the control group compared to all other groups (Figure 6b; F_4,20_ = 4.272, *p* = 0.0117), while it was similar between the SteLL-treated and Sham groups and lower in the fluoxetine-treated group. No significant changes were observed among control, Sham, and treatment groups IL-6 (Figure 6c; F_4,20_ = 0.9077, *p* = 0.4784), IL-17 (Figure 6e; F_4,20_ = 1.959, *p* = 0.1399), interferon (IFN) γ (Figure 6f; F_4,20_ = 0.9162, *p* = 0.4738), or tumor necrosis factor (TNF) α (Figure 6g; F_4,20_ = 0.6219, *p* = 0.6522). Regarding the anti-inflammatory cytokine IL-10, both doses of SteLL increased its release, with the 4 mg/kg dose showing a more significant effect compared to the control. Fluoxetine did not alter IL-10 levels (Figure 6d; F_4,20_ = 6.209, *p* = 0.0020), and IL-10 was not elevated in the Sham group relative to the control.

### 2.6. SteLL Ameliorated Monoaminergic Signaling Following Reserpine Treatment

**Figure 7:**
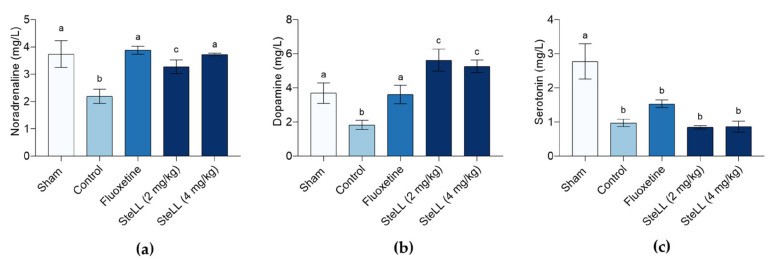
Effects of *Schinus terebinthifolia* leaf lectin (SteLL) on the levels of the monoamines noradrenaline (**a**), dopamine (**b**), and serotonin (**c**) in the brain of reserpine-treated mice. Reserpine-treated animals received intraperitoneal injections of PBS (control), fluoxetine (10 mg/kg) or SteLL (2 or 4 mg/kg). The Sham group did not receive reserpine administration and was treated with PBS i.p. Data are expressed as mean ± SEM (n = 5). Bars labeled with different letters represent statistically significant differences between groups (*p* < 0.05), whereas bars sharing the same letter indicate no significant difference (*p* > 0.05), as determined by ANOVA followed by Bonferroni post hoc test.

Treatments with SteLL or fluoxetine significantly increased noradrenaline levels compared to the control (Figure 7a; F_4,20_ = 6.040, *p* = 0.0024). Noradrenaline levels in the SteLL 4 mg/kg and fluoxetine groups were similar to those in the sham group. Dopamine levels were significantly elevated in all treated groups compared to control, with SteLL (2 and 4 mg/kg) inducing a stronger response than fluoxetine (Figure 7b; F_4,20_ = 8.924, *p* = 0.0003) and also reaching levels higher than those observed in the Sham group. Serotonin levels were not significantly affected by SteLL or fluoxetine treatment (Figure 7c; F_4,20_ = 10.48, *p* < 0.0001). As expected, the Sham group exhibited higher levels of the three monoamines than the control group.

## 3. Discussion

Depression and anxiety continue to affect a growing proportion of the global population, with profound consequences for public health systems, productivity, and overall quality of life. In this context, the exploration of natural products as therapeutic alternatives has garnered increasing attention. Among these, plant lectins such as SteLL have demonstrated promising pharmacological properties, including anxiolytic and antidepressant-like effects. In these reports, Lima et al. [13,14] did not employ protocols for anxiety or depression induction, whereas the work by Lima et al. [15] investigated these effects in a chronic stress-induced model. The present study expands upon this evidence by evaluating whether SteLL maintains its neurobehavioral effects in a pharmacological model of monoaminergic depletion induced by reserpine and by exploring the potential mechanisms underlying its effects.

The use of animal models remains fundamental for elucidating the neurobiological substrates of emotional disorders and for identifying novel therapeutic agents [18]. The reserpine model is particularly relevant in this context, as it simulates core features of depressive-like states by irreversibly blocking the vesicular monoamine transporter-2 (VMAT-2), resulting in the depletion of key monoamines (serotonin, dopamine, and noradrenalin) through enhanced degradation by monoamine oxidase [19,20,21]. These monoamines play critical roles in modulating cognition, emotion, motivation, and social behavior [22,23,24]. Disruption of monoaminergic signaling often leads to the emergence of depressive and anxiety-like phenotypes [25].

Our results showed that SteLL significantly reversed anxiety-like behavior in reserpine-treated mice. Typically, rodents avoid the open arms of EPM due to a natural aversion to open spaces; therefore, increased time spent in the open arms is indicative of reduced anxiety [26]. Furthermore, crossing data from the OFT and the number of entries into the open arms of the EPM showed no significant changes in general locomotor activity. This suggests that the increased time in the open arms reflects an anxiolytic effect of SteLL rather than being attributable to sedation [27].

In the TST, the data suggest that SteLL neither affected the animals’ initial motivation to escape nor altered the pattern or frequency of transitions between active and passive coping strategies. However, SteLL significantly reduced the total immobility time, indicating that the animals spent less overall time in a passive, despair-like state [28]. Together, these findings suggest a specific antidepressant-like effect of SteLL that promotes sustained active coping behavior without changing the onset or frequency of stress-induced behavioral responses.

Importantly, these effects were accompanied by modulation of brain monoamine levels—specifically dopamine and norepinephrine –suggesting that SteLL’s mechanism of action in this model may be independent of the serotonergic system. This finding is particularly relevant given the limited efficacy of selective serotonin reuptake inhibitors (SSRIs) in certain populations, such as individuals with treatment-resistant depression [29,30]. The fact that SteLL did not significantly alter serotonin levels strengthens the hypothesis that its effects are preferentially mediated via dopaminergic and noradrenergic pathways, similarly to a variety of commercial antidepressants (such as reboxetine—a selective inhibitor of noradrenaline [31]—and bupropion—a selective inhibitor of noradrenaline and dopamine [32]).

Changes in body composition are commonly observed in individuals with mood disorders and may result from both the condition itself and pharmacological interventions [33,34]. Animals treated with SteLL did not exhibit weight loss, a side effect observed in fluoxetine treatment. Furthermore, at the lower dose, SteLL-treated animals maintained a higher body weight compared to the Sham group. These findings suggest that SteLL may help prevent some of the metabolic disturbances often linked to depression, preserving energy balance during depressive episodes, unlike certain antidepressant therapies that may exacerbate weight loss. Supporting this hypothesis, the similar relative water and food intake observed across groups indicates that the effects of SteLL and fluoxetine are likely mediated by metabolic or physiological mechanisms rather than differences in consumption.

Neuroinflammatory modulation also appears to be a key mechanism underlying SteLL’s effects. Behavioral alterations induced by reserpine were accompanied by elevated levels of the pro-inflammatory cytokine IL-2, aligning with growing evidence that links inflammation to the pathophysiology of depression [35,36,37,38]. Microglia respond to different stimuli by adopting either a pro-inflammatory (M1) or anti-inflammatory (M2) phenotype, with M1 releasing cytokines like IL-2, TNF-α, IL-1β, IL-6, and IL-12, and M2 producing factors such as IL-10, TGF-β, and BDNF [38]. An increase in IL-4 levels, an anti-inflammatory cytokine, in the control group compared to the Sham group likely reflects a compensatory response to the elevated IL-2 levels, attempting to counteract the inflammation caused by reserpine. Although slightly lower than in the control group, IL-4 levels in animals treated with SteLL remained equivalent to those in the Sham group. It is important to consider that these SteLL-treated animals no longer exhibited the pro-inflammatory state induced by reserpine, as indicated by the low IL-2 levels.

SteLL treatment significantly reduced IL-2 levels and increased the release of IL-10, an anti-inflammatory cytokine associated with M2 microglial activation and neuroprotection [39]. This selective cytokine modulation suggests that SteLL may help restore balance in the neuroimmune axis, potentially reestablishing homeostasis and reducing neuronal damage induced by inflammation. The more pronounced increase in IL-10 at the higher dose indicates a dose-dependent immunomodulatory effect, reinforcing the therapeutic potential of this molecule. Increased IL-10 levels have been associated with improvements in depressive-like behavior in studies involving physical exercise alone [37], exercise combined with cognitive-behavioral therapy [40], or direct administration of the cytokine [41].

The immunomodulatory properties of SteLL likely derive from its capacity to bind glycan structures on the surface of immune cells [17], which may, in turn, influence cytokine secretion profiles related to different glial cell phenotypes. Whether this interaction also extends to neuronal cells, altering monoaminergic signaling directly or indirectly, remains an open and intriguing question.

Given the dual effects observed—monoaminergic and immunomodulatory –, we propose two non-mutually exclusive hypotheses regarding the mechanism of action of SteLL: (1) SteLL may interact with glycan structures located on or near monoamine receptors, thereby modulating their function and enhancing the bioavailability of dopamine and norepinephrine; or (2) it may bind to other membrane-associated glycoconjugates, triggering intracellular signaling cascades that promote the synthesis or release of these monoamines. Further studies, including receptor-binding assays and glycomics-based approaches, will be necessary to validate these hypotheses.

The investigation of the behavioral and neurobiological effects of the lectin SteLL followed a progressive approach, starting from simple models and advancing to more complex and pathophysiologically relevant experimental conditions. Initially, the anxiolytic and antidepressant effects of SteLL were observed in animals without any induction of emotional disorders, serving as a preliminary screening to assess its psychotropic potential and suggesting the involvement of monoaminergic pathways and the carbohydrate recognition domain in its effects [13,14]. Subsequently, study using a non-pharmacological chronic stress model allowed for the evaluation of the SteLL’s efficacy in a context closer to environmental conditions that trigger anxiety and depression, revealing that SteLL acts broadly by modulating monoamines, oxidative stress, and inflammatory cytokines [15]. Here, the use of the pharmacological model induced by reserpine represented a significant advance in understanding SteLL’s mechanism of action, as it demonstrated its ability to reverse biochemical and behavioral changes typical of depression with a known organic basis. The relevance of the pharmacological model induced by reserpine lies in the fact that it poses a more rigorous therapeutic challenge, simulating a depressive state with a well-established cause (a clear and direct failure in neurotransmission), allowing validation of SteLL’s efficacy in conditions with severe impairment of monoaminergic neurotransmission, in a real and specific pathological scenario.

The observation that SteLL increased serotonin and noradrenaline levels, but not dopamine, in a chronic stress model [15], while in the reserpine model it increased dopamine and noradrenaline but not serotonin, suggests a differential modulatory action of the lectin on monoaminergic systems, possibly dependent on the pathological context and mechanisms involved in each model and subsequently, each individual. In the chronic stress model, neurochemical dysfunction tends to be multifactorial, involving gradual and compensatory changes in the serotonergic and noradrenergic systems, which are strongly affected by prolonged stress [42,43]. The absence of effect on serotonin may indicate that SteLL preferentially acts on pathways related to the stress response and mood regulation mediated by dopamine and noradrenaline. On the other hand, the reserpine model induces a direct and acute depletion of monoamines—primarily dopamine and noradrenaline, and to a lesser extent, serotonin [44]. In this scenario, SteLL’s effect in restoring dopamine and noradrenaline, but not serotonin, may reflect a specific ability of the lectin to compensate for the loss of these two monoamines directly linked to reserpine action, possibly through modulation of synthesis, release, or reuptake of these neurotransmitters, as well as influence on brain inflammation. This selectivity may also point to different molecular mechanisms and signaling pathways activated by SteLL in each context, including its interactions with specific receptors, enzymes, and monoamine transport systems.

In summary, these differences indicate that SteLL has a flexible and adaptive modulatory profile, capable of selectively acting on the monoaminergic systems most relevant to the underlying pathology, which reinforces its potential as a therapeutic agent for affective disorders with different etiologies. Finally, this study underscores the importance of employing multiple behavioral models to evaluate the robustness and reproducibility of antidepressant-like effects. Combining pharmacological models (such as reserpine-induced) with stress-based models (e.g., chronic unpredictable stress) enables a more comprehensive assessment of candidate compounds and helps establish their translational relevance.

It is important to note that this study was conducted exclusively in male mice. This approach was chosen to minimize variability associated with hormonal cycles, maintain consistency with previous studies in the field, and reduce the overall number of animals used. However, now that positive results have been obtained, this limitation can be addressed in future research. Affective disorders show well-documented sex-specific prevalence and neurobiological differences, highlighting the need for inclusive study designs. Therefore, future studies should incorporate female cohorts to investigate potential sex-dependent effects and enhance the translational relevance of the findings.

Finally, the effects of SteLL observed in the reserpine-induced model suggest potential relevance for the treatment of mood disorders in humans. However, it is important to recognize that model—primarily its emphasis on monoaminergic depletion—does not capture the full complexity of human depression, which involves genetic, environmental, and neuroendocrine components. To strengthen translational relevance, future research should incorporate complementary preclinical models that better reflect the multifactorial nature of depression, alongside early-phase clinical studies to assess the safety, pharmacokinetics, and efficacy of SteLL. These steps are essential for advancing SteLL as a therapeutic candidate, particularly for treatment-resistant forms of depression.

## 4. Materials and Methods

### 4.1. Lectin Purification

After obtaining authorization (no. 72024) from the *Instituto Chico Mendes de Conservação da Biodiversidade*, leaves of *S. terebinthifolia* were collected on the campus of the *Universidade Federal de Pernambuco* (UFPE), Recife, Brazil (8°02′59.4”S 34°56′53.3”W). A representative specimen is deposited at the *Instituto Agronômico de Pernambuco* (Recife) under reference number 73431. Additionally, this study is registered in the *Sistema Nacional de Gestão do Patrimônio Genético e do Conhecimento Tradicional Associado* under code A37C1E4.

The extraction and purification of the SteLL protein were conducted following the protocol described by Gomes et al. [16]. Briefly, collected leaves were washed with distilled water and air-dried for three days at 28 °C. The dried leaves were then ground into a fine powder using a multiprocessor (LQL-4 multiprocessor, Metvisa, Brusque, Brazil). This powder was homogenized in 0.15 M NaCl (10% *w*/*v*) for 16 h at 4 °C. The extract was obtained by centrifugation (15 min, 3500× *g*, 4 °C) and subsequently subjected to affinity chromatography using a chitin column (7.5 × 1.5 cm; Sigma-Aldrich, St. Louis, MO, USA). The lectin was eluted with 1.0 M acetic acid, and its purity was confirmed by SDS-PAGE [45]. Finally, the purified lectin was lyophilized using a LIOTOP L101 freeze-dryer (Liobras, São Carlos, Brazil) and stored at –20 °C.

Protein concentration was determined using the method described by Lowry et al. [46], with a bovine serum albumin standard curve ranging from 31.25 to 500 µg/mL. The hemagglutinating activity (HA) assay, performed to confirm SteLL’s carbohydrate-binding capacity, followed the protocol outlined by Procópio et al. [47]. This assay was conducted one day prior to the experiment. HA (units) was defined as the reciprocal of the highest SteLL dilution capable of inducing complete erythrocyte agglutination. Specific HA (units/mg) was calculated as the ratio of HA to protein concentration. Glutaraldehyde-fixed rabbit erythrocytes were used, collected as approved by the Ethics Committee of UFPE (process no. 23076.033782/2015-70)

### 4.2. Animals

For the in vivo experiments, twenty-five male Swiss mice (20–25 g), aged 4–6 weeks, were used. All animals were obtained from the Instituto Keizo Asami vivarium at UFPE. The mice were housed in cages (maximum of six animals per cage) at a controlled temperature of 24 °C, maintained on a 12 h light/dark cycle, with free access to food and water. Sample size was defined based on previous works [13,14,15]. All procedures were approved in advance by the Ethics Committee on Animal Use of UFPE (protocol no. 0010/2021).

### 4.3. Reserpine Protocol and Treatments

The animals were randomly assigned to five groups, each containing six mice. For ten consecutive days, animals received daily intraperitoneal injections of either fluoxetine (10 mg/kg, positive control) or SteLL (2 or 4 mg/kg), administered immediately before reserpine (0.2 mg/kg, i.p.), except the Sham group, which received phosphate-buffered saline (PBS, 10 mL/kg). SteLL doses were defined as previously described by Lima et al. [15] and shown to be non-toxic [11]. To minimize confounding variables, all treatments were administered in a fixed sequence, starting with the Sham group, followed by control, positive control, and SteLL-treated groups. Group allocation at each stage was known only to the researcher responsible for administering the treatments. Neurobehavioral tests (described below) were conducted after the treatment period.

### 4.4. Open Field Test (OFT)

The apparatus consisted of a box measuring 40 cm (length) × 50 cm (width) × 60 cm (height), with the floor divided into four equal quadrants by marked lines. The number of crossings (defined as the animal fully entering a new quadrant with all four paws) and rearings (defined as the animal standing on its hind paws, a behavior associated with exploration or escape attempts) were recorded [48]. The test lasted 5 min. Quantitative changes in crossings and rearings were interpreted as indicators of locomotor activity and anxiety-related behaviors, respectively, according to Hernández-León et al. [49].

### 4.5. Elevated Plus Maze (EPM) Test

The Elevated Plus Maze (EPM) apparatus consisted of two open arms (50 × 10 cm) and two closed arms (50 × 10 cm, with 40 cm-high walls), arranged in a plus-shape and elevated 50 cm above the floor. The arms were connected by a central square platform (10 × 10 cm). Each animal was placed individually in the central area and allowed to explore the maze for 5 min. The number of entries and the time spent in both the open and closed arms were recorded. Avoidance of the open arms and/or increased time spent in the closed arms were interpreted as indicators of anxiety-like behavior [50].

### 4.6. Tail Suspension Test (TST)

Each animal was individually suspended by the tail, approximately 1 cm from the tip, using adhesive tape and held 50 cm above the ground. During a 5 min session, the following parameters were recorded: immobility time (total duration of inactivity), number of immobility episodes (frequency of immobile periods), and latency to the first immobility episode (time elapsed before the animal became immobile for the first time) [51,52]. Animals exhibiting prolonged immobility were considered to display depression-like behavior.

### 4.7. Body Weight and Chemical Parameters

#### 4.7.1. Monitoring of Body Weight and Consumption of Food and Water

Body weight as well as water and food intake were monitored daily from the first day of reserpine administration through the final day of treatment with lectin or fluoxetine. The total observation period lasted 24 days, consisting of 10 days of reserpine administration followed by 14 days of treatment. At the end of the protocol, body weight change was calculated for each group as the difference between the mean final body weight and the mean initial body weight. Relative values of water and food consumption were calculated as: food consumed (g) or water consumed (mL)/body weight (g) × 100.

#### 4.7.2. Brain Dissection

After the behavioral tests, animals were euthanized by intraperitoneal injection of ketamine (100 mg/kg) and xylazine (10 mg/kg). The brains were removed by craniotomy, sectioned, and placed in 2 mL of PBS with a protease inhibitor cocktail (Sigma-Aldrich), then homogenized using a vortex mixer. The whole-brain homogenates were diluted with PBS to normalize protein concentrations across all samples.

#### 4.7.3. Cytokine Quantification

Cytokine levels in brain homogenates were measured using the BD Cytometric Bead Array Mouse Th1/Th2/Th17 kit (BD Biosciences, Franklin Lakes, NJ, USA), following the manufacturer’s instructions. The panel quantified interferon-gamma (IFN-γ), interleukins (IL-2, IL-4, IL-6, IL-10, IL-17), and tumor necrosis factor-alpha (TNF-α). Samples were analyzed on a BD Accuri C6 flow cytometer (BD Biosciences), and cytokine concentrations were calculated from standard curves ranging from 0 to 5000 pg/mL.

#### 4.7.4. Monoamine Quantification

Levels of dopamine, noradrenaline, and serotonin in brain homogenates were determined using High-Performance Liquid Chromatography with ultraviolet detection (HPLC-UV). For sample preparation, 1 mL of homogenate was passed through a Clean-Up^®^ Endcapped C18 column (500 mg/6 mL; United Chemical Technology, Bristol, PA, USA), which had been preconditioned with a solution of 0.11% phosphoric acid and acetonitrile (95:5, *v*/*v*). The resulting eluate was filtered through a 0.22 µm membrane before analysis. Chromatographic separation was performed using an HPLC system fitted with an HP 1050 Diode Array Detector (Shimadzu Corp., Tokyo, Japan). Separation was achieved by two reverse-phase columns connected in series: a Zorbax C8 column (4.6 × 150 mm, 3.5 µm; Agilent Technologies, Santa Clara, CA, USA) followed by a Shim-pack C18 column (4.6 × 250 mm, 5 µm; Shimadzu). The mobile phase consisted of 0.11% phosphoric acid and acetonitrile (95:5, *v*/*v*) and was delivered at a flow rate of 1 mL/min. Detection was set at a wavelength of 280 nm, and injection volume was 50 µL. Calibration curves for dopamine, noradrenaline, and serotonin (Sigma-Aldrich) were linear within a concentration range of 1 to 20 mg/L. All solvents and reagents used were chromatographic grade.

### 4.8. Statistical Analysis

Comparisons between experimental and control groups were conducted using one-way analysis of variance (ANOVA) followed by Bonferroni post hoc tests. Each animal represented a replicate, and thus each group was tested in quintuplicate. A *p*-value of less than 0.05 was considered statistically significant. All statistical analyses were performed using GraphPad Prism software version 8.0 (La Jolla, CA, USA).

## 5. Conclusions

SteLL demonstrates significant antidepressant- and anxiolytic-like effects in a pharmacological model of monoaminergic depletion, likely mediated through dopaminergic and noradrenergic modulation as well as anti-inflammatory mechanisms. These findings highlight SteLL as a promising candidate for developing novel therapeutics, especially for individuals who exhibit inadequate responses to serotonergic drugs. Importantly, these pharmacological results provide scientific support for the traditional use of *S. terebinthifolia* leaves in the treatment of neuropsychological disorders. Future research should explore the molecular interactions between SteLL and neuronal and glial membranes, along with its long-term safety and efficacy.

## Data Availability

Data are contained within the article.
